# Methyl 3-(4-chloro­phen­yl)-1-methyl-1,2,3,3a,4,11c-hexa­hydro­benzo[*f*]chromeno[4,3-*b*]pyrrole-3a-carboxyl­ate

**DOI:** 10.1107/S1600536810005465

**Published:** 2010-02-13

**Authors:** B. Gunasekaran, S. Kathiravan, R. Raghunathan, V. Manivannan

**Affiliations:** a Department of Physics, AMET University, Kanathur, Chennai 603 112, India; b Department of Organic Chemistry, University of Madras, Guindy Campus, Chennai 600 025, India; c Department of Research and Development, PRIST University, Vallam, Thanjavur 613 403, Tamil Nadu, India

## Abstract

In the title compound, C_24_H_22_ClNO_3_, the dihedral angle between the naphthalene ring system and the chloro­phenyl ring is 67.44 (4)°. The pyrrolidine and dihydro­pyran rings exhibit envelope and half chair conformations, respectively. In the crystal structure, weak C—H⋯π inter­actions are observed.

## Related literature

For the biological activity of chromenopyrrole derivatives, see: Caine (1993 ▶); Tidey (1992 ▶); Carlson (1993 ▶); Sokoloff *et al.* (1990 ▶); Wilner (1985 ▶); Sobral & Rocha Gonsalves (2001*a* ▶,*b* ▶); Brockmann & Tour (1995 ▶); Suslick *et al.* (1992 ▶); Di Natale *et al.* (1998 ▶). For related structures, see: Nirmala *et al.* (2009*a* ▶,*b* ▶); Gunasekaran *et al.* (2009 ▶). For puckering parameters, see: Cremer & Pople (1975 ▶). For asymmetry parameters, see: Nardelli (1983 ▶).
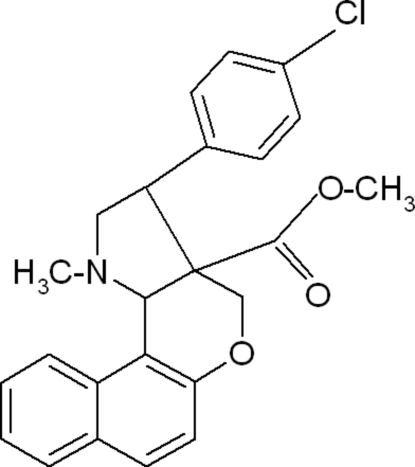


## Experimental

### 

#### Crystal data

C_24_H_22_ClNO_3_
*M* *_r_* = 407.88
Monoclinic, 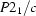

*a* = 12.6951 (8) Å
*b* = 19.8829 (13) Å
*c* = 8.0799 (6) Å
β = 106.396 (4)°
*V* = 1956.6 (2) Å^3^
*Z* = 4
Mo *K*α radiation
μ = 0.22 mm^−1^
*T* = 295 K
0.20 × 0.20 × 0.20 mm

#### Data collection

Bruker Kappa APEXII diffractometer
Absorption correction: multi-scan (*SADABS*; Sheldrick, 1996 ▶) *T*_min_ = 0.954, *T*_max_ = 0.957
18246 measured reflections
4759 independent reflections
3643 reflections with *I* > 2σ(*I*)
*R*_int_ = 0.039

#### Refinement

*R*[*F* ^2^ > 2σ(*F* ^2^)] = 0.042
*wR*(*F* ^2^) = 0.119
*S* = 1.05
4759 reflections
264 parameters
H-atom parameters constrained
Δρ_max_ = 0.26 e Å^−3^
Δρ_min_ = −0.31 e Å^−3^

### 

Data collection: *APEX2* (Bruker, 2004 ▶); cell refinement: *SAINT* (Bruker, 2004 ▶); data reduction: *SAINT*; program(s) used to solve structure: *SHELXS97* (Sheldrick, 2008 ▶); program(s) used to refine structure: *SHELXL97* (Sheldrick, 2008 ▶); molecular graphics: *PLATON* (Spek, 2009 ▶); software used to prepare material for publication: *SHELXL97*.

## Supplementary Material

Crystal structure: contains datablocks I. DOI: 10.1107/S1600536810005465/is2523sup1.cif

Structure factors: contains datablocks I. DOI: 10.1107/S1600536810005465/is2523Isup2.hkl

Additional supplementary materials:  crystallographic information; 3D view; checkCIF report

## Figures and Tables

**Table 1:** Hydrogen-bond geometry (Å, °) *Cg*1, *Cg*2 and *Cg*3 are the centroids of the C1–C5/C10, C5–C10 and C16–C21 rings, respectively.

*D*—H⋯*A*	*D*—H	H⋯*A*	*D*⋯*A*	*D*—H⋯*A*
C17—H17⋯*Cg*2^i^	0.93	2.99	3.615 (6)	126
C18—H18⋯*Cg*1^i^	0.93	2.75	3.637 (5)	159
C20—H20⋯*Cg*3^ii^	0.93	2.89	3.651 (9)	139

Symmetry codes: (i) 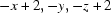; (ii) 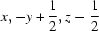.

**Fig. 1.**
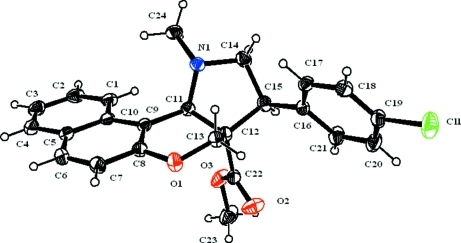
The molecular structure of (I), with atom labels and 30% probability displacement ellipsoids for non-H atoms.

## supplementary crystallographic information

### Comment

Chromenopyrrole compounds are used in the treatment of impulsive disorders (Caine, 1993), aggressiveness (Tidey, 1992), parkinson's disease (Carlson, 1993), psychoses, memory disorders (Sokoloff *et al.*, 1990), anxiety and depression (Wilner, 1985). Pyrroles are also very useful precursors in porphyrin synthesis (Sobral & Rocha Gonsalves, 2001*a*, *b*), and as monomers for polymer chemistry (Brockmann & Tour, 1995), with applications ranging from non linear optical materials (Suslick *et al.*, 1992) to electronic noses (Di Natale *et al.*, 1998).

The geometric parameters of the title molecule (Fig. 1) agree well with reported similar structure (Nirmala *et al.*, 2009*a*, *b*; Gunasekaran *et al.*, 2009). The dihedral angle between the naphthalene ring system and the chlorophenyl ring is 67.44 (4)°. The pyrrolidine ring [N1/C14/C15/C12/C11] exhibits an envelope conformation with envelope on C11 with an asymmetry parameter (Nardelli, 1983) ΔC_s_ (C11) = 4.05 (3) and with the puckering parameters (Cremer & Pople, 1975) q2 = 0.4275 (2) Å and φ2 = 214.69 (6)°. The six-membered heterocyclic ring [C8/C9/C11/C12/C13/O1] of the benzochromenopyrrole moiety adopts a half-chair conformation with the puckering parameters Q = 0.4697 (2) Å, Θ = 132.51 (3)° and φ = 82.84 (5)°. The sum of bond angles around N1 [332.44 (12)°] indicate the *sp*^3^ hybridized state of atom N1 in the molecule.

The crystal packing is stabilized by weak intermolecular C—H···π [C17—H17···*Cg*2(2-*x*, -*y*, 2-*z*), C18—H18···*Cg*1(2-*x*, -*y*, 2-*z*) and C20—H20···*Cg*3(*x*, 1/2-*y*, -1/2+*z*); Table 1] interactions. *Cg*1, *Cg*2 and *Cg*3 are the centroids of the rings C1–C5/C10, C5–C10 and C16–C21, respectively.

### Experimental

A mixture of (*Z*)-methyl 2-((1-formylnaphthalen-2-yloxy) methyl) -3-(4-chloro phenylacrylate (20 mmol) and sarcosine (30 mmol) were refluxed in benzene for 20 h and the solvent was removed under reduced pressure. The crude product was subjected to column chromatography to get the pure product. Chloroform and methanol (1:1) solvent mixture was used for the crystallization under slow evaporation method.

### Refinement

H atoms were positioned geometrically and refined using riding model, with C—H = 0.93 Å and *U*_iso_(H) = 1.2*U*_eq_(C) for aromatic C—H, C—H = 0.98 Å and *U*_iso_(H) = 1.2*U*_eq_(C) for C—H, C—H = 0.97 Å and *U*_iso_(H) = 1.2*U*_eq_(C) for CH_2_, and C—H = 0.96 Å and *U*_iso_(H) = 1.5*U*_eq_(C) for CH_3_.

### Crystal data

**Table:**

C_24_H_22_ClNO_3_	*F*(000) = 856
*M**_r_* = 407.88	*D*_x_ = 1.385 Mg m^−^^3^
Monoclinic, *P*2_1_/*c*	Mo *K*α radiation, λ = 0.71073 Å
Hall symbol: -P 2ybc	Cell parameters from 7245 reflections
*a* = 12.6951 (8) Å	θ = 2.6–28.0°
*b* = 19.8829 (13) Å	µ = 0.22 mm^−^^1^
*c* = 8.0799 (6) Å	*T* = 295 K
β = 106.396 (4)°	Block, colourless
*V* = 1956.6 (2) Å^3^	0.20 × 0.20 × 0.20 mm
*Z* = 4	

### Data collection

**Table:**

Bruker Kappa APEXII diffractometer	4759 independent reflections
Radiation source: fine-focus sealed tube	3643 reflections with *I* > 2σ(*I*)
graphite	*R*_int_ = 0.039
Detector resolution: 0 pixels mm^-1^	θ_max_ = 28.3°, θ_min_ = 1.7°
ω and φ scans	*h* = −16→16
Absorption correction: multi-scan (*SADABS*; Sheldrick, 1996)	*k* = −25→26
*T*_min_ = 0.954, *T*_max_ = 0.957	*l* = −10→10
18246 measured reflections	

### Refinement

**Table:**

Refinement on *F*^2^	Primary atom site location: structure-invariant direct methods
Least-squares matrix: full	Secondary atom site location: difference Fourier map
*R*[*F*^2^ > 2σ(*F*^2^)] = 0.042	Hydrogen site location: inferred from neighbouring sites
*wR*(*F*^2^) = 0.119	H-atom parameters constrained
*S* = 1.05	*w* = 1/[σ^2^(*F*_o_^2^) + (0.0498*P*)^2^ + 0.5781*P*] where *P* = (*F*_o_^2^ + 2*F*_c_^2^)/3
4759 reflections	(Δ/σ)_max_ = 0.012
264 parameters	Δρ_max_ = 0.26 e Å^−^^3^
0 restraints	Δρ_min_ = −0.31 e Å^−^^3^

### Fractional atomic coordinates and isotropic or equivalent isotropic displacement parameters (Å^2^)

**Table:**

	*x*	*y*	*z*	*U*_iso_*/*U*_eq_	
C1	−0.41608 (12)	0.03370 (9)	0.79567 (19)	0.0390 (3)	
H1	−0.3911	0.0740	0.8513	0.047*	
C2	−0.51822 (13)	0.01074 (10)	0.7918 (2)	0.0475 (4)	
H2	−0.5618	0.0359	0.8439	0.057*	
C3	−0.55817 (14)	−0.04982 (11)	0.7110 (2)	0.0528 (5)	
H3	−0.6270	−0.0655	0.7118	0.063*	
C4	−0.49560 (14)	−0.08579 (10)	0.6309 (2)	0.0493 (4)	
H4	−0.5228	−0.1258	0.5757	0.059*	
C5	−0.39005 (13)	−0.06350 (8)	0.63013 (18)	0.0383 (3)	
C6	−0.32741 (14)	−0.09955 (8)	0.5409 (2)	0.0441 (4)	
H6	−0.3544	−0.1397	0.4864	0.053*	
C7	−0.22864 (14)	−0.07645 (8)	0.5336 (2)	0.0431 (4)	
H7	−0.1889	−0.1001	0.4721	0.052*	
C8	−0.18609 (12)	−0.01636 (7)	0.61941 (19)	0.0350 (3)	
C9	−0.24015 (11)	0.01983 (7)	0.71585 (17)	0.0309 (3)	
C10	−0.34747 (11)	−0.00277 (7)	0.71656 (17)	0.0326 (3)	
C11	−0.18799 (11)	0.08273 (7)	0.80899 (17)	0.0304 (3)	
H11	−0.2444	0.1169	0.8038	0.036*	
C12	−0.09886 (11)	0.11107 (7)	0.73256 (18)	0.0313 (3)	
C13	−0.02720 (12)	0.05300 (7)	0.7070 (2)	0.0359 (3)	
H13A	0.0299	0.0702	0.6600	0.043*	
H13B	0.0081	0.0327	0.8180	0.043*	
C14	−0.05563 (13)	0.13099 (9)	1.0426 (2)	0.0445 (4)	
H14A	0.0127	0.1192	1.1273	0.053*	
H14B	−0.0935	0.1641	1.0929	0.053*	
C15	−0.03273 (12)	0.15937 (7)	0.87920 (19)	0.0355 (3)	
H15	−0.0684	0.2035	0.8577	0.043*	
C16	0.08658 (11)	0.16971 (7)	0.88440 (18)	0.0328 (3)	
C17	0.17038 (12)	0.12687 (7)	0.97076 (19)	0.0359 (3)	
H17	0.1539	0.0902	1.0303	0.043*	
C18	0.27803 (13)	0.13754 (8)	0.97024 (19)	0.0393 (3)	
H18	0.3333	0.1083	1.0284	0.047*	
C19	0.30196 (13)	0.19206 (8)	0.8823 (2)	0.0420 (4)	
C20	0.22150 (15)	0.23519 (9)	0.7951 (2)	0.0526 (4)	
H20	0.2385	0.2715	0.7348	0.063*	
C21	0.11452 (14)	0.22398 (8)	0.7978 (2)	0.0479 (4)	
H21	0.0599	0.2537	0.7400	0.057*	
C22	−0.14094 (12)	0.15066 (8)	0.56664 (19)	0.0365 (3)	
C23	−0.28508 (17)	0.21789 (11)	0.3981 (3)	0.0661 (6)	
H23A	−0.2616	0.2636	0.4238	0.099*	
H23B	−0.3638	0.2159	0.3664	0.099*	
H23C	−0.2595	0.2017	0.3043	0.099*	
C24	−0.18914 (13)	0.05770 (8)	1.10856 (19)	0.0406 (3)	
H24A	−0.1412	0.0525	1.2232	0.061*	
H24B	−0.2307	0.0172	1.0745	0.061*	
H24C	−0.2383	0.0946	1.1063	0.061*	
N1	−0.12435 (10)	0.07114 (6)	0.99011 (15)	0.0346 (3)	
O1	−0.08863 (9)	0.00275 (6)	0.59363 (15)	0.0432 (3)	
O2	−0.08968 (11)	0.16075 (8)	0.46603 (17)	0.0627 (4)	
O3	−0.24040 (10)	0.17653 (7)	0.54843 (17)	0.0550 (3)	
Cl1	0.43761 (4)	0.20640 (3)	0.88362 (8)	0.07301 (19)	

### Atomic displacement parameters (Å^2^)

**Table:**

	*U*^11^	*U*^22^	*U*^33^	*U*^12^	*U*^13^	*U*^23^
C1	0.0372 (8)	0.0499 (9)	0.0318 (7)	0.0016 (7)	0.0126 (6)	0.0035 (6)
C2	0.0370 (8)	0.0716 (12)	0.0374 (8)	0.0018 (8)	0.0162 (6)	0.0084 (8)
C3	0.0387 (9)	0.0759 (13)	0.0453 (9)	−0.0137 (9)	0.0146 (7)	0.0119 (9)
C4	0.0474 (9)	0.0571 (11)	0.0416 (9)	−0.0176 (8)	0.0096 (7)	0.0057 (8)
C5	0.0408 (8)	0.0416 (8)	0.0315 (7)	−0.0065 (6)	0.0086 (6)	0.0050 (6)
C6	0.0538 (10)	0.0364 (8)	0.0421 (8)	−0.0101 (7)	0.0134 (7)	−0.0065 (7)
C7	0.0510 (9)	0.0381 (8)	0.0441 (9)	−0.0018 (7)	0.0197 (7)	−0.0099 (7)
C8	0.0371 (7)	0.0349 (7)	0.0361 (7)	−0.0015 (6)	0.0154 (6)	−0.0034 (6)
C9	0.0344 (7)	0.0309 (7)	0.0293 (6)	0.0001 (6)	0.0122 (5)	0.0004 (5)
C10	0.0345 (7)	0.0372 (7)	0.0269 (6)	−0.0009 (6)	0.0101 (5)	0.0050 (5)
C11	0.0327 (7)	0.0298 (7)	0.0320 (7)	0.0022 (5)	0.0147 (5)	−0.0022 (5)
C12	0.0302 (7)	0.0318 (7)	0.0342 (7)	−0.0005 (5)	0.0130 (5)	−0.0039 (6)
C13	0.0335 (7)	0.0369 (8)	0.0412 (8)	−0.0008 (6)	0.0169 (6)	−0.0086 (6)
C14	0.0430 (8)	0.0553 (10)	0.0380 (8)	−0.0093 (7)	0.0161 (7)	−0.0149 (7)
C15	0.0347 (7)	0.0332 (7)	0.0392 (8)	−0.0005 (6)	0.0112 (6)	−0.0075 (6)
C16	0.0350 (7)	0.0282 (7)	0.0348 (7)	−0.0031 (5)	0.0091 (6)	−0.0028 (5)
C17	0.0419 (8)	0.0316 (7)	0.0350 (7)	−0.0006 (6)	0.0123 (6)	0.0041 (6)
C18	0.0374 (8)	0.0406 (8)	0.0390 (8)	0.0028 (6)	0.0094 (6)	0.0001 (6)
C19	0.0357 (8)	0.0456 (9)	0.0457 (9)	−0.0091 (7)	0.0129 (6)	−0.0045 (7)
C20	0.0534 (10)	0.0429 (9)	0.0603 (11)	−0.0119 (8)	0.0139 (8)	0.0153 (8)
C21	0.0426 (9)	0.0363 (8)	0.0586 (10)	−0.0013 (7)	0.0042 (7)	0.0133 (7)
C22	0.0373 (8)	0.0358 (7)	0.0378 (7)	−0.0062 (6)	0.0129 (6)	−0.0039 (6)
C23	0.0531 (11)	0.0644 (13)	0.0772 (14)	0.0042 (9)	0.0127 (10)	0.0339 (11)
C24	0.0484 (9)	0.0450 (9)	0.0325 (7)	0.0021 (7)	0.0180 (6)	−0.0012 (6)
N1	0.0367 (6)	0.0390 (7)	0.0302 (6)	0.0013 (5)	0.0127 (5)	−0.0045 (5)
O1	0.0407 (6)	0.0439 (6)	0.0530 (6)	−0.0066 (5)	0.0262 (5)	−0.0194 (5)
O2	0.0641 (8)	0.0837 (10)	0.0493 (7)	0.0096 (7)	0.0309 (6)	0.0169 (7)
O3	0.0418 (6)	0.0609 (8)	0.0655 (8)	0.0096 (6)	0.0203 (6)	0.0279 (6)
Cl1	0.0447 (3)	0.0830 (4)	0.0973 (4)	−0.0185 (2)	0.0299 (3)	−0.0060 (3)

### Geometric parameters (Å, °)

**Table:**

C1—C2	1.367 (2)	C14—N1	1.466 (2)
C1—C10	1.417 (2)	C14—C15	1.537 (2)
C1—H1	0.9300	C14—H14A	0.9700
C2—C3	1.395 (3)	C14—H14B	0.9700
C2—H2	0.9300	C15—C16	1.5174 (19)
C3—C4	1.360 (3)	C15—H15	0.9800
C3—H3	0.9300	C16—C21	1.386 (2)
C4—C5	1.413 (2)	C16—C17	1.387 (2)
C4—H4	0.9300	C17—C18	1.384 (2)
C5—C6	1.410 (2)	C17—H17	0.9300
C5—C10	1.423 (2)	C18—C19	1.376 (2)
C6—C7	1.352 (2)	C18—H18	0.9300
C6—H6	0.9300	C19—C20	1.367 (2)
C7—C8	1.410 (2)	C19—Cl1	1.7425 (16)
C7—H7	0.9300	C20—C21	1.383 (2)
C8—O1	1.3653 (17)	C20—H20	0.9300
C8—C9	1.3776 (19)	C21—H21	0.9300
C9—C10	1.4361 (19)	C22—O2	1.1930 (18)
C9—C11	1.5126 (19)	C22—O3	1.3325 (19)
C11—N1	1.4765 (18)	C23—O3	1.444 (2)
C11—C12	1.5406 (18)	C23—H23A	0.9600
C11—H11	0.9800	C23—H23B	0.9600
C12—C22	1.516 (2)	C23—H23C	0.9600
C12—C13	1.5198 (19)	C24—N1	1.4520 (18)
C12—C15	1.5714 (19)	C24—H24A	0.9600
C13—O1	1.4289 (18)	C24—H24B	0.9600
C13—H13A	0.9700	C24—H24C	0.9600
C13—H13B	0.9700		
			
C2—C1—C10	121.25 (16)	N1—C14—H14A	110.3
C2—C1—H1	119.4	C15—C14—H14A	110.3
C10—C1—H1	119.4	N1—C14—H14B	110.3
C1—C2—C3	121.08 (16)	C15—C14—H14B	110.3
C1—C2—H2	119.5	H14A—C14—H14B	108.6
C3—C2—H2	119.5	C16—C15—C14	117.09 (13)
C4—C3—C2	119.50 (15)	C16—C15—C12	114.85 (11)
C4—C3—H3	120.3	C14—C15—C12	103.47 (12)
C2—C3—H3	120.3	C16—C15—H15	106.9
C3—C4—C5	121.27 (17)	C14—C15—H15	106.9
C3—C4—H4	119.4	C12—C15—H15	106.9
C5—C4—H4	119.4	C21—C16—C17	117.57 (14)
C6—C5—C4	121.01 (15)	C21—C16—C15	119.16 (13)
C6—C5—C10	119.41 (14)	C17—C16—C15	123.26 (13)
C4—C5—C10	119.55 (15)	C18—C17—C16	121.46 (14)
C7—C6—C5	120.90 (15)	C18—C17—H17	119.3
C7—C6—H6	119.6	C16—C17—H17	119.3
C5—C6—H6	119.6	C19—C18—C17	118.97 (14)
C6—C7—C8	119.81 (15)	C19—C18—H18	120.5
C6—C7—H7	120.1	C17—C18—H18	120.5
C8—C7—H7	120.1	C20—C19—C18	121.23 (15)
O1—C8—C9	123.95 (13)	C20—C19—Cl1	119.60 (13)
O1—C8—C7	113.48 (12)	C18—C19—Cl1	119.17 (13)
C9—C8—C7	122.55 (14)	C19—C20—C21	119.02 (15)
C8—C9—C10	117.61 (13)	C19—C20—H20	120.5
C8—C9—C11	119.69 (12)	C21—C20—H20	120.5
C10—C9—C11	122.64 (12)	C20—C21—C16	121.75 (15)
C1—C10—C5	117.32 (13)	C20—C21—H21	119.1
C1—C10—C9	123.12 (14)	C16—C21—H21	119.1
C5—C10—C9	119.54 (13)	O2—C22—O3	122.82 (15)
N1—C11—C9	113.84 (11)	O2—C22—C12	124.48 (14)
N1—C11—C12	101.31 (11)	O3—C22—C12	112.61 (12)
C9—C11—C12	111.78 (11)	O3—C23—H23A	109.5
N1—C11—H11	109.9	O3—C23—H23B	109.5
C9—C11—H11	109.9	H23A—C23—H23B	109.5
C12—C11—H11	109.9	O3—C23—H23C	109.5
C22—C12—C13	110.44 (12)	H23A—C23—H23C	109.5
C22—C12—C11	115.41 (11)	H23B—C23—H23C	109.5
C13—C12—C11	108.19 (12)	N1—C24—H24A	109.5
C22—C12—C15	109.24 (12)	N1—C24—H24B	109.5
C13—C12—C15	110.73 (11)	H24A—C24—H24B	109.5
C11—C12—C15	102.58 (10)	N1—C24—H24C	109.5
O1—C13—C12	112.27 (12)	H24A—C24—H24C	109.5
O1—C13—H13A	109.1	H24B—C24—H24C	109.5
C12—C13—H13A	109.1	C24—N1—C14	111.13 (12)
O1—C13—H13B	109.1	C24—N1—C11	115.39 (12)
C12—C13—H13B	109.1	C14—N1—C11	105.92 (12)
H13A—C13—H13B	107.9	C8—O1—C13	116.81 (11)
N1—C14—C15	106.93 (12)	C22—O3—C23	116.61 (14)
			
C10—C1—C2—C3	0.6 (2)	N1—C14—C15—C12	2.04 (15)
C1—C2—C3—C4	−1.7 (3)	C22—C12—C15—C16	−84.34 (15)
C2—C3—C4—C5	0.9 (3)	C13—C12—C15—C16	37.50 (17)
C3—C4—C5—C6	−177.35 (16)	C11—C12—C15—C16	152.74 (12)
C3—C4—C5—C10	0.9 (2)	C22—C12—C15—C14	146.84 (12)
C4—C5—C6—C7	176.87 (16)	C13—C12—C15—C14	−91.33 (14)
C10—C5—C6—C7	−1.4 (2)	C11—C12—C15—C14	23.91 (14)
C5—C6—C7—C8	1.5 (3)	C14—C15—C16—C21	−144.79 (15)
C6—C7—C8—O1	−176.79 (15)	C12—C15—C16—C21	93.52 (17)
C6—C7—C8—C9	1.7 (3)	C14—C15—C16—C17	35.9 (2)
O1—C8—C9—C10	173.56 (13)	C12—C15—C16—C17	−85.78 (17)
C7—C8—C9—C10	−4.7 (2)	C21—C16—C17—C18	−0.3 (2)
O1—C8—C9—C11	−3.6 (2)	C15—C16—C17—C18	179.04 (13)
C7—C8—C9—C11	178.12 (14)	C16—C17—C18—C19	0.2 (2)
C2—C1—C10—C5	1.2 (2)	C17—C18—C19—C20	−0.5 (2)
C2—C1—C10—C9	179.29 (14)	C17—C18—C19—Cl1	179.12 (12)
C6—C5—C10—C1	176.35 (14)	C18—C19—C20—C21	0.8 (3)
C4—C5—C10—C1	−1.9 (2)	Cl1—C19—C20—C21	−178.75 (14)
C6—C5—C10—C9	−1.8 (2)	C19—C20—C21—C16	−0.9 (3)
C4—C5—C10—C9	179.94 (14)	C17—C16—C21—C20	0.7 (3)
C8—C9—C10—C1	−173.33 (13)	C15—C16—C21—C20	−178.69 (16)
C11—C9—C10—C1	3.7 (2)	C13—C12—C22—O2	−36.0 (2)
C8—C9—C10—C5	4.7 (2)	C11—C12—C22—O2	−159.04 (15)
C11—C9—C10—C5	−178.21 (13)	C15—C12—C22—O2	86.05 (18)
C8—C9—C11—N1	−94.21 (15)	C13—C12—C22—O3	147.42 (13)
C10—C9—C11—N1	88.77 (16)	C11—C12—C22—O3	24.34 (18)
C8—C9—C11—C12	19.84 (18)	C15—C12—C22—O3	−90.57 (15)
C10—C9—C11—C12	−157.18 (13)	C15—C14—N1—C24	−154.95 (13)
N1—C11—C12—C22	−159.78 (11)	C15—C14—N1—C11	−28.92 (15)
C9—C11—C12—C22	78.63 (15)	C9—C11—N1—C24	−72.57 (15)
N1—C11—C12—C13	75.95 (13)	C12—C11—N1—C24	167.29 (12)
C9—C11—C12—C13	−45.64 (15)	C9—C11—N1—C14	164.04 (11)
N1—C11—C12—C15	−41.11 (13)	C12—C11—N1—C14	43.90 (13)
C9—C11—C12—C15	−162.70 (11)	C9—C8—O1—C13	16.3 (2)
C22—C12—C13—O1	−67.74 (15)	C7—C8—O1—C13	−165.30 (14)
C11—C12—C13—O1	59.45 (15)	C12—C13—O1—C8	−45.08 (18)
C15—C12—C13—O1	171.13 (11)	O2—C22—O3—C23	0.4 (3)
N1—C14—C15—C16	−125.40 (13)	C12—C22—O3—C23	177.05 (15)

### Hydrogen-bond geometry (Å, °)

**Table:**

Cg1, Cg2 and Cg3 are the centroids of the C1–C5/C10, C5–C10 and C16–C21 rings, respectively.

**Table:**

*D*—H···*A*	*D*—H	H···*A*	*D*···*A*	*D*—H···*A*
C17—H17···Cg2^i^	0.93	2.99	3.615 (6)	126
C18—H18···Cg1^i^	0.93	2.75	3.637 (5)	159
C20—H20···Cg3^ii^	0.93	2.89	3.651 (9)	139

Symmetry codes: (i) −*x*+2, −*y*, −*z*+2; (ii) *x*, −*y*+1/2, *z*−1/2.
